# Neuroprogression in post-traumatic stress disorder: a systematic review

**DOI:** 10.47626/2237-6089-2020-0099

**Published:** 2021-10-22

**Authors:** Thyago Antonelli-Salgado, Luis Francisco Ramos-Lima, Cristiane dos Santos Machado, Ryan Michael Cassidy, Taiane de Azevedo Cardoso, Flávio Kapczinski, Ives Cavalcante Passos

**Affiliations:** 1 Departamento de Psiquiatria Escola de Medicina Universidade Federal do Rio Grande do Sul Porto Alegre RS Brazil Programa de Pós-Graduação em Psiquiatria e Ciências do Comportamento, Departamento de Psiquiatria, Escola de Medicina, Universidade Federal do Rio Grande do Sul, Porto Alegre, RS, Brazil.; 2 Centro de Pesquisa Experimental Centro de Pesquisa Clínica Hospital de Clínicas de Porto Alegre Porto Alegre RS Brazil Laboratório de Psiquiatria Molecular, Centro de Pesquisa Experimental (CPE), Centro de Pesquisa Clínica (CPC), Hospital de Clínicas de Porto Alegre (HCPA), Porto Alegre, RS, Brazil.; 3 Instituto Nacional de Ciência e Tecnologia Translacional em Medicina Porto Alegre RS Brazil Instituto Nacional de Ciência e Tecnologia Translacional em Medicina (INCT-TM), Porto Alegre, RS, Brazil.; 4 McGovern Medical School University of Texas Health Science Center at Houston Houston TX USA McGovern Medical School, University of Texas Health Science Center at Houston, Houston, TX, USA.; 5 Mood Disorders Program Department of Psychiatry and Behavioral Neuroscience McMaster University Hamilton ON Canada Mood Disorders Program, Department of Psychiatry and Behavioral Neuroscience, McMaster University, Hamilton, ON, Canada.

**Keywords:** Neuroprogression, PTSD, neuroimaging

## Abstract

**Introduction:**

Neuroprogression has been proposed as the pathological rewiring of the brain that takes place in parallel with clinical and neurocognitive deterioration in the course of psychiatric disorders. This study aims to review the biological underpinnings and clinical outcomes related to neuroprogression in post-traumatic stress disorder (PTSD).

**Methods:**

We performed a systematic review by searching PubMed, Embase, and Web of Science for articles published between January 1, 1960, and January 6, 2020. Inclusion criteria were met when articles assessed brain changes, neurocognition, functioning, inflammation, oxidative stress, and neurotrophins in patients with PTSD. Narrative review articles, case reports, and preclinical studies were excluded.

**Results:**

A total of 965 abstracts were identified and 15 articles were included in our systematic review. It seems that for a subset of patients whose symptoms worsen or are maintained at a high intensity there is a progressive change in the frontal lobe, especially the prefrontal cortex, and worsening of both neurocognition (verbal memory and facial recognition) and functioning (physical, psychological, social and environmental).

**Conclusion:**

Although current findings associate progressive reduction in frontal lobe size with neurocognitive impairment, further research is needed to characterize PTSD as a neuroprogressive disorder.

## Introduction

Post-traumatic stress disorder (PTSD) has a lifetime prevalence of 3.9%.^1^ PTSD is considered a debilitating condition that develops from exposure to traumatic events such as actual or threatened death, actual or threatened serious injury, or actual or threatened sexual violence. The DSM-5^2^ lists 20 diagnostic criteria for PTSD divided into four clusters of symptoms: re-experience of the traumatic event; avoidance; persistent negative thoughts or feelings; and trauma-related arousal and reactivity.

It is known that the clinical course of PTSD is not uniform and may vary depending upon the type of trauma, whether the patient was pediatric, adult, or elderly at the time of the inciting trauma, whether they were responsible or not for the trauma, and so on.^3^ For instance, a prospective cohort study of World Trade Center first-responders found several subtypes of symptom trajectories over 12 years; 76% had no symptoms, 12% had worsening symptoms, 7.5% had improving symptoms, and 4.5% had chronic symptoms.^4^ Additionally, a 4-year prospective longitudinal epidemiological study of 125 young adults with DSM-IV-diagnosed PTSD found that only half recovered to the point of no longer meeting diagnostic criteria.^5^ PTSD symptoms can persist for several years. A 25-year longitudinal study found that Vietnam veterans continue to report PTSD symptoms that impair functioning 40 years after the war, with 16% of veterans who served in the Vietnam War having worsening of symptoms over time.^6^

The consequences of PTSD are numerous; this diagnosis leads to very serious interpersonal and occupational challenges and has been estimated to result in 3.6 days of lost productivity per month.^7^ War veterans with PTSD have extensive functional impairments such as unemployment and income disparities,^8^ family and relationship difficulties,^9^ aggressive behavior,^10^ and poor quality of life.^11^ PTSD is associated with reduced quality of life in various domains such as physical, psychological, social, and environmental.^12^ Moreover, PTSD is associated with an increased rate of suicide attempts.^13^

It is also known that PTSD has especially strong associations with various immune^14^ and cardiovascular^15^ diseases. It has been suggested that stress-related changes in the endocrine and immune systems are involved in the pathophysiological processes linking PTSD and somatic comorbidity.^16^

PTSD symptoms and brain changes can play an important role in maintaining the disorder. A primary component of the symptomatology of PTSD is re-experiencing or reliving of the traumatic memory, which has elements of both psychophysiological reactivation and psychological distress.^17^ A unique part of this condition is the repeated reactivation of the traumatic memory and the associated stress response with the attendant risk of progressive augmentation of the individual’s reactivity.^18^ It has been suggested that there is a failure of the retention and extinction of conditioned fear in PTSD.^19^ Rauch et al.^20^ have suggested that an exaggerated amygdala response underpins the excessive acquisition of fear associations and the expression of fear responses observed in PTSD. A corresponding deficit of frontal cortical functioning plays a central role in mediating extinction. There is also a deficit of appreciation of the context of safety, which is related to hippocampal function.

Evidence from neuroimaging studies has suggested areas of the brain that may be damaged by psychological trauma. A meta-analysis^21^ included 66 MRI studies and showed that compared with non-traumatized or traumatized control subjects, patients with PTSD were found to have reduced brain volume, intracranial volume, and hippocampus, insula, and anterior cingulate volumes. Other reviews have shown structural brain changes such as reduced hippocampal volume^22^ and changes in white matter volume in frontal regions and the cingulum.^23^ Functional brain changes have also been shown in a previous review, the main replicated finding showed increased amygdala activation in participants with PTSD.^22^ Despite these findings, it is unclear whether brain changes can be progressive over time after a diagnosis of PTSD.

Several studies have addressed the concept of neuroprogression in psychiatric disorders, including bipolar disorder,^24^ schizophrenia,^25^ and major depressive disorder.^26^ This concept has been proposed as the pathological rewiring of the brain that takes place in parallel with neurocognitive deterioration in the course of some psychiatric disorders.^24^ Researchers have found that at least a subgroup of patients with these disorders has a pernicious course with cognitive deterioration.^24^ Moreover, a review showed the potential biological underpinnings of neuroprogression in PTSD, including changes in oxidative stress, inflammatory markers, and cortisol regulation.^27^ However, no systematic review has yet evaluated the outcomes associated with neuroprogression. Therefore, our systematic review aims to assess neuroprogression in PTSD through the following parameters: brain anatomy, neurocognition, functioning, inflammation, oxidative stress, and neurotrophins.

## Methods

### Guidelines

The present study was registered with PROSPERO (CRD42019135609). We followed the guidelines described by the Preferred Reporting Items for Systematic Reviews and Meta-Analysis (PRISMA) statement.^28^

### Search strategy

We searched PubMed, Embase, and Web of Science with the following search terms: (“PTSD” OR “Stress Disorder, Post Traumatic” OR “Stress Disorders, Post Traumatic” OR “Neuroses, Posttraumatic” OR “Posttraumatic Neuroses” OR “Posttraumatic Stress Disorders” OR “Posttraumatic Stress Disorder” OR “Stress Disorder, Posttraumatic” OR “Stress Disorders, Posttraumatic” OR “Neuroses, Post-Traumatic” OR “Neuroses, Post Traumatic” OR “Post-Traumatic Neuroses” OR “Post-Traumatic Stress Disorders” OR “Post Traumatic Stress Disorders” OR “Post-Traumatic Stress Disorder” OR “Stress Disorder, Post-Traumatic” OR “Chronic Post-Traumatic Stress Disorder” OR “Chronic Post Traumatic Stress Disorder” OR “Delayed Onset Post-Traumatic Stress Disorder” OR “Delayed Onset Post Traumatic Stress Disorder” OR “Acute Post-Traumatic Stress Disorder” OR “Acute Post Traumatic Stress Disorder”) AND (“neuroprogression” OR “staging” OR “illness progression” OR “progression” OR “allostatic load”) for literature published from January 1, 1960 to January 6, 2020. Medical Subject Heading (MeSH) terms were used in PubMed. Retrieved abstracts were loaded into EndNote online to automatically remove duplicates. We also searched the reference lists of included studies. Two researchers (TAS and CSM) independently screened and selected titles and abstracts for full-text inclusion, with disagreements mediated by LFRL who made the final decision in such cases. When available, we sought out translated versions of articles in languages that none of the authors spoke. We did not search the grey literature (evidence not published in commercial publications).

### Selection criteria

We included articles that assessed the following neuroprogression-related outcomes in patients with PTSD: brain changes, neurocognition, inflammation, oxidative stress, and neurotrophins. We selected longitudinal studies that assessed such parameters over time or cross-sectional and meta-analysis studies that related such parameters to illness duration. We excluded preclinical studies and narrative review articles. If two or more studies reported the same clinical outcome within the same dataset, we included only the most recent.

### Quality assessment

Two researchers (TAS and LFRL) rated each article with the Newcastle-Ottawa Quality Assessment Scale (NOQAS) to evaluate the risk of bias and quality of the study.^29^ A third researcher (ICP) resolved scoring disagreements. The quality assessment of these studies can be found in Table S1 (available as online-only supplementary material).

### Data extraction

Each article was reviewed, and the information extracted was compiled into an Excel workbook. The following variables were extracted: first author; publication year; country; psychiatric diagnosis; diagnostic tool; severity scales; study design; type of neuroimaging study; brain region assessed; scales used to assess cognition and functioning; number of patients in each group; gender; mean age; duration of illness, age of onset, medications status; race; and major depressive disorder (MDD) comorbidity. We assessed MDD comorbidity because several studies included patients with both PTSD and MDD.

## Results

**Figure 1:**
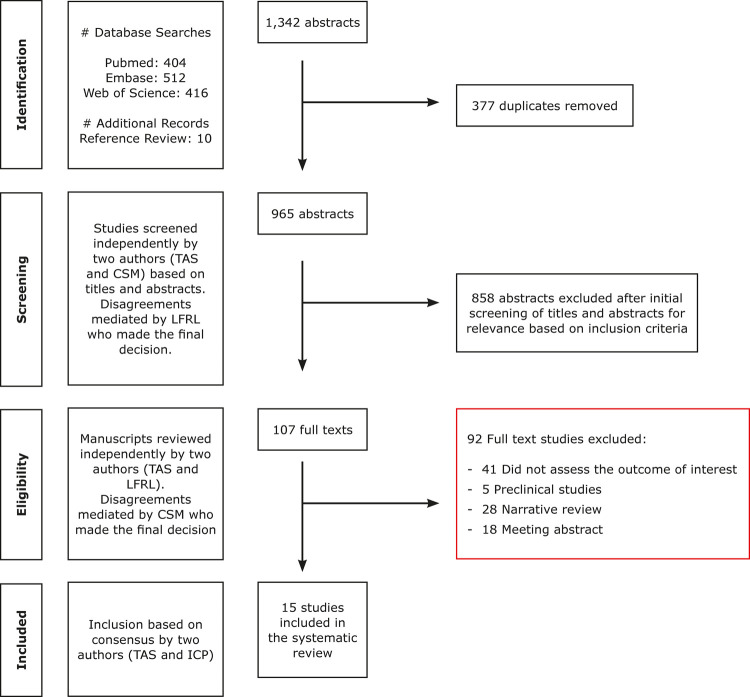
Flowchart of review process and study selection

Figure 1 illustrates the study selection process. We found 965 unique abstracts. A total of 107 studies were included in the eligibility phase; however, only 15 studies met the full criteria for inclusion in our systematic review. Characteristics of the included studies are described in Tables 1 to 3.

### Brain changes

Table 1 shows the 10 studies that assessed brain changes.

**Table 1:** Characteristics of included studies

	Outcomes	Types of Trauma	Study design	Diagnostic Criteria	Illness duration in months ± SD	Type of neuroimaging study	N (PTSD, non-PTSD)	Male (%)		Mean age in years ± SD		MDD Comorbidity (PTSD%/ non-PTSD%)

								PTSD	non-PTSD	PTSD	Non-PTSD	
Heyn, 2019^30^	PFC, precentral gyrus, amygdala, hippocampus	Mixed	Cohort	DSM-IV	--	MRI 3-T fMRI	48 (27/21)*	33%	23%	14.01±2.81	13.92±2.44	44%/--
Yoon, 2017^31^	PFC, amygdala, hippocampus	Subway fire Disaster	Cohort	DSM-IV	1.65	MRI 3-TDTI	59 (30/29)*	36.7	38.0	27	26.4	--/--
Keding, 2015^32^	vm-PFC, ACC	Mixed	Cross-sectional	DSM-IV	46±36	MRI 3-T	54 (27/27)*	33%	52%	14.2±2.7	13.6±3.0	70%/--
Chao, 2014^33^	Hippocampus, caudate nucleus	War trauma	Cross-sectional	DSM-IV	200.4±104.4	MRI 1.5-T	55 (55/0)^†^	90%	--	44.9±8.9	--	--/--
Cardenas, 2011^34^	Whole brain volume	Mixed	Cohort	DSM-IV	372±72	MRI 1.5-T	47 (25/22)^‡^	100%	100%	50.5±6.6	52.1±5.6	--/--
Felmingham, 2009^35^	GMV of the hippocampus and ACC	Mixed	Cross-sectional	ICD-10	66	MRI 1.5-T	38 (21/17)^†^	NA	NA	NA	NA	52%/0%
Hakamata, 2007^36^	GMV of the right OFC	Breast cancer	Cohort	DSM-IV	--	MRI 1.5-T	76 (9/67)^†^	100%	100%	45.6±6.2	47.1±5.7	78%/21%
Villarreal, 2002^37^	Hippocampus, CSF total WMV, total GMV	Mixed	Cross-sectional	DSM-IV	--	MRI 1.5-T	22 (12/10)*	17%	20%	43±9.3	44±11.4	42%/--
Bonne, 2001^38^	Hippocampus, amygdala	Mixed	Cohort	DSM-IV	--	MRI 2-T	37 (10/27)^†^	30%	56%	33.7±8.9	29.8±10.1	--/--
De Bellis, 1999^39^	Cerebral and intracranial volume	Mixed	Cross-sectional	DSM-IV DSM-III-R	33.6±25.2	MRI 1.5-T	105 (44/61)*	57%	59%	12.2±2.4	12.0±2.3	45%/0%

* Trauma-unexposed controls; ^†^ trauma-exposed controls; ^‡^ part of the controls had been exposed to trauma.

ACC = anterior cingulate cortex; DSM = Diagnostic and Statistical Manual of Mental Disorders; fMRI = functional MRI; ICD = International Classification of Diseases; GMV = grey matter volume; MDD = major depressive disorder; m-PFC = medial prefrontal cortex; MRI = magnetic resonance imaging; OFC = orbitofrontal cortex; PCC = posterior cingulate cortex; PFC = prefrontal cortex; PTSD = post-traumatic stress disorder; vl = ventrolateral; vm = ventromedial.

The first, Heyn 2019, is a one-year follow-up of 27 patients with PTSD and 21 trauma-unexposed controls. This study showed that youths with PTSD exhibited sustained reductions in grey matter volume in the right ventromedial prefrontal cortex (PFC) and bilateral ventrolateral PFC. Additionally, group-by-time analyses revealed aberrant longitudinal development in the dorsolateral PFC. Furthermore, patients with PTSD exhibited atypical longitudinal decreases in ventromedial PFC-amygdala and ventrolateral PFC-hippocampus connectivity. Finally, volumetric abnormalities in the ventromedial PFC and ventrolateral PFC were predictive of PTSD symptom severity.^30^

The second study, Yoon 2017, is a five-year follow-up study of 30 patients with PTSD and 29 trauma-unexposed controls without trauma. This study showed that patients with PTSD developed a strengthening of amygdala-insula connection, had unaffected connectivity of the amygdala-PFC, and lower amygdala connections with the thalamus and hippocampus. All demonstrated improvements in PTSD symptoms over time, and this correlated with normalization of the amygdala connections with all of these regions except for the amygdala-hippocampus connection, which remained low. The strength of the amygdala-PFC connectivity was inversely associated with PTSD symptom severity.^31^

The third study, Keding 2015, is a cross-sectional study of 27 medication-free pediatric patients with PTSD and 27 trauma-unexposed matched controls. This study showed that PTSD youth had reduced volume in the anterior ventromedial PFC, which inversely correlated with PTSD duration. In this group, re-experiencing symptoms inversely correlated with subgenual anterior cingulate cortex and right anterior hippocampus grey matter volume. However, no group differences were observed for the amygdala.^32^

The fourth study, Chao 2014, is a cross-sectional study of 55 patients with chronic PTSD and no matched control subjects that assessed the relationship between illness duration and volumes of the hippocampus and the caudate nucleus (which served as a “control” region). There was a significant negative correlation between right hippocampal volume and PTSD duration.^33^

The fifth study, Cardenas 2011, is a two-year follow-up study of 25 patients with PTSD and 22 controls (17 trauma-exposed), all males, which assessed changes in brain volume using deformation morphometry. Patients with PTSD did not show significant ongoing brain atrophy compared to patients without. Patients with PTSD were then subgrouped into those with increasing or decreasing symptoms; those with worsening symptomatology showed accelerated atrophy throughout the brain, particularly brainstem and frontal and temporal lobes.^34^ Conversely, those whose symptoms improved did not have accelerated atrophy except in a small left parietal region.^34^

The sixth study, Felmingham 2009, is a cross-sectional study of 21 patients with PTSD and 17 trauma-exposed controls of unrecorded sex that showed a significant negative correlation between right hippocampal volume and PTSD duration. These authors also found that patients with PTSD presented significant reductions in the rostral anterior cingulate cortex, but this finding had no association with illness duration.^35^

The seventh study, Hakamata 2007, is a two-year follow-up study of 9 patients with PTSD and 67 trauma-exposed controls, all males, that showed the grey matter volume of the right orbitofrontal cortex was significantly smaller in cancer survivors with PTSD than in cancer survivors without PTSD or healthy subjects.^36^ However, the interaction between the diagnosis and the timing of the right orbitofrontal cortex’s grey matter volume measurement was not significant.^36^

The eighth study, Villarreal 2002, is a cross-sectional study with 12 patients with PTSD and 10 trauma-unexposed controls that showed that subjects with PTSD had smaller absolute and normalized bilateral hippocampal volumes, besides less cerebrospinal fluid and white matter. It found negative correlations between total Clinician-Administered PTSD Scale (CAPS) scores and left hippocampal volumes and between the re-experiencing subscale of the CAPS and left hippocampal volumes. However, hippocampal volumes did not correlate with duration of illness.^37^

The ninth study, Bonne 2001, is a six-month follow-up study of 10 patients with PTSD and 27 trauma-exposed controls that showed no reduction in hippocampal and amygdala volume in the PTSD subjects between 1 week and 6 months.^38^

The tenth study, De Bellis 1999, is a cross-sectional study of 44 maltreated children and adolescents with PTSD and 61 trauma-unexposed matched controls that showed that PTSD subjects had smaller intracranial and cerebral volumes than matched controls. Brain volume robustly and positively correlated with the age of onset of PTSD trauma and negatively correlated with duration of trauma. However, they found no difference in hippocampal volume between the two groups.^39^

### Neurocognitive changes and functioning

Table 2 shows the two studies about neurocognition and one study about functioning.

**Table 2:** Characteristics of included studies

	Outcome	Types of trauma	Study design	Diagnostic criteria	Illness duration in months ± SD	Neurocognitive and functioning scales	N (PTSD, non-PTSD)	Male (%)		Mean age in years ± SD		MDD Comorbidity (PTSD%/ non-PTSD%)

								PTSD	non-PTSD	PTSD	Non-PTSD	
Cardenas, 2011^34^	Cognition	Mixed	Cohort	DSM-IV	372±72	CVLT, Faces I and II, Family Pictures I and II, WMS-III (Digit Span and Spatial Span)	47 (25/22)*	100%	100%	50.5±6.6	52.1±5.6	-- / --
Emdad, 2005^40^	Cognition	War trauma	Cross-sectional	DSM-IV	--	BDT, RSPM, BVRT	40 (23/17)^†^	100%	100%	38.65±6.23	37.88± 8.58	0% / 0%
Bryant, 2015^12^	Functioning	Mixed	Cohort	DSM-IV	72	WHOQOL-Bref,	1,084 (791/293)^‡^	--	--	--	--	-- / --

* Part of the controls was exposed to trauma; ^†^ trauma-unexposed controls; ^‡^ trauma-exposed controls.

BDT = Block Design Test; BVRT = Benton Visual Retention Test; CVLT = California Verbal Learning Test; DSM = Diagnostic and Statistical Manual of Mental Disorders; MDD = major depressive disorder; PTSD = post-traumatic stress disorder; RSPM = Raven’s Standard Progressive Matrices; WHOQOL-Bref = World Health Organization Quality of Life; WMS = Wechsler Memory Scale.

The first study that assessed neurocognition, Cardenas 2011, was described in the previous section. In addition to the anatomical features described, this study showed that a higher rate of brain atrophy was associated with a higher rate of decline in verbal memory and with delayed facial recognition.^34^

The second, Emdad 2005, is a cross-sectional study of 23 patients with PTSD and 17 trauma-unexposed controls, all males, which showed that PTSD subjects had lower scores for short-term visual memory and general intelligence than controls. These impairments were negatively associated with the duration of the trauma.^40^

The study that assessed functioning, Bryant 2015, is a six-year follow-up of 1084 patients who had experienced a traumatic injury. Included subjects had different trajectories of symptoms and functionality (physical, psychological, social, and environmental). A subgroup of 73% of the sample maintained subsyndromic PTSD and was used as controls in the analyses. Compared to this group, authors found that: 1) 4% maintained high intensity of symptoms with high impairment of functioning; 2) 6% exhibited high intensity of symptoms at baseline, but these gradually decreased, as opposed to functioning, which was initially reduced but improved after 6 years; 3) 8% displayed a pattern of relatively low initial symptoms of PTSD at baseline that increased over the subsequent 24 months before returning to their initial level by the 6-year measurement, but functioning was lower as symptoms worsened and partially increased after symptoms improved; 4) the remaining 10% of the sample exhibited relatively low levels of PTSD symptoms at baseline, but these gradually increased over the years, as opposed to functioning (physical, psychological, social, and environmental), which deteriorated after 6 years.^12^

### Inflammation

Table 3 shows the 3 studies that assessed inflammation.

**Table 3:** Characteristics of included studies

	Inflammatory markers assessed	Types of trauma	Study design	Diagnostic criteria	Illness duration in months ± SD	Blood fraction	N (PTSD, non-PTSD)	Male (%)		Mean age in years ± SD		MDD Comorbidity (PTSD%/ non-PTSD%)

								PTSD	non-PTSD	PTSD	Non-PTSD	
Passos, 2015^41^	IL-1β, IL-2, IL-2R IL-4, IL-6, IL-6R, IL-8, IL-10, INF-γ, TNF-α, CRP	Mixed	Meta-analysis	--	--	--	--	--	--	--	--	-- / --
Vidovic, 2011^42^	IL-6, TNF-alfa	War trauma	Cohort	DSM-IV and ICD-10	--	Serum	64 (39/25)*	100%	100%	38.5±9.1	32.6±8.6	0%/0%
Spivak, 1997^43^	IL-1β, IL-2R	War trauma	Cross-sectional	DSM-III-R	90±82.8	Serum	38 (19/19)*	100%	100%	25.3±10.9	31.7±10.4	0%/0%

* Trauma-unexposed controls.

CRP = C-reactive protein; DSM = Diagnostic and Statistical Manual of Mental Disorders; ICD = International Classification of Diseases; IL-1β = interleukin-1β; IL-2 = interleukin-2; IL-2R = interleukin-2 receptor; IL-4 = Interleukin-4; IL-6 = interleukin-6; IL-6R = interleukin-6 receptor; IL-8 = interleukin-8; IL-10 = interleukin-10; INF-γ = interferon-gamma; MDD = major depressive disorder; PTSD = post-traumatic stress disorder; TNF-α = tumor necrosis factor-alpha.

The first, Passos 2015, is a meta-analysis including 20 studies. This meta-analysis aimed to describe cytokine levels in PTSD and demonstrated that interleukin-6, interleukin-1β, interferon-γ, and tumor necrosis factor were significantly higher in patients with PTSD, as compared to controls.^41^ Besides, illness duration was positively associated with interleukin-1β levels, but no significant association was noted for interleukin-6 or tumor necrosis factor.^41^

The second study, Vidovic 2011, is a five-year follow-up study of 39 patients with PTSD and 25 trauma-unexposed controls, all males, that showed a significant difference in TNF-α serum concentration in PTSD patients at the first assessment. However, the difference was no longer significant at a second assessment after 5 years. No differences were found in IL-6 either at the first or the second assessment.^42^

The third study, Spivak 1997, is a cross-sectional study of 19 male patients with combat-related PTSD and 19 trauma-unexposed controls. This study showed that serum interleukin-1β levels were significantly higher in the PTSD patients,^43^ while serum interleukin-2 receptor levels did not differ between the two groups. Interleukin-1β levels showed a significant positive correlation with duration of PTSD symptoms.^43^

## Discussion

This is the first systematic review to assess progressive brain changes and neurocognitive decline in patients with PTSD in addition to evaluating other parameters such as inflammatory pattern, oxidative stress, and neurotrophins according to the concept of neuroprogression. Prior reviews have focused on single timepoint data, which limits the ability to provide conclusive findings regarding neuroprogression. Based on our review, we conclude that this is a relatively untapped field for PTSD; only a few studies have directly addressed our research questions.

Our first conclusion relates to the demonstrated anatomical changes that occur secondary to PTSD. The studies included in our systematic review demonstrated brain changes in the frontal lobe, especially the PFC, in both adult and pediatric patients. In adults, the studies showed that throughout the period of illness, there was a volumetric reduction in white matter of the frontal lobe and grey matter of the dorsolateral PFC.^34^ A volumetric reduction over time was also observed in the anterior cingulate cortex by a longitudinal study,^34^ but a cross-sectional study found no association between the volumetric reduction of this region and illness duration.^35^ No interaction was found between time and volumetric reduction in the orbitofrontal cortex grey matter.^36^

However, there was a volumetric reduction in other regions such as the brainstem, the caudate and thalamus, and temporal and occipital grey matter including, specifically, insula, anterior temporal lobe, and extrastriatal cortex.^34^

Three selected studies evaluated the influence of PTSD on pediatric brain development and reported unique findings that add nuance to the concept of neuroprogression. A longitudinal study that assessed adolescents with PTSD found reductions in the ventromedial and ventrolateral PFC (including associated fMRI findings of reduced ventromedial/ventrolateral - PFC connectivity with the amygdala and hippocampus, respectively), but an increase in the dorsolateral PFC. Control youths demonstrated an expected decrease in dorsolateral PFC size, which is believed to be associated with synaptic pruning.^30^ These data provide a unique perspective on the pathological rewiring aspect of neuroprogression, as these adolescents experienced altered neurocircuit development with preservation of an immature brain anatomy, rather than only experiencing reductions in size.

In line with this finding, two cross-sectional studies found a negative correlation between illness duration and reduction of total cerebral volume^39^ and, more specifically, anterior ventromedial PFC volume.^32^ Another previous review^44^ had shown that childhood maltreatment exerts a prepotent influence on brain development. According to this review, maltreatment is associated with reliable morphological alterations in anterior cingulate, dorsal lateral prefrontal and orbitofrontal cortex, corpus callosum, and adult hippocampus, and with enhanced amygdala response to emotional faces and diminished striatal response to anticipated rewards.

Changes in the hippocampus and amygdala have been reported in PTSD.^45^ Six selected articles have evaluated these regions. There were conflicting findings regarding hippocampus volume. While some studies have shown progression of hippocampal volume reduction with illness duration,^33,35^ others have not confirmed these findings.^34,37-39^ There was no association between changes in amygdala volume and follow-up time.^34,38^ According to some of the authors, negative results may be associated with a short follow-up or short illness duration.^33,34^ A recent prospective cohort study with 247 war veterans exposed to trauma assessed post-traumatic stress symptoms and brain changes for 24 years and showed that an increase in symptomatology was associated with smaller hippocampal volume.^46^ We did not include this study in our systematic review because most of the included participants did not meet the criteria for PTSD diagnosis.

The strength of amygdala-PFC connectivity was inversely associated with PTSD symptom severity, but changes in connections improved in parallel with improved symptoms.^31^ The m-PFC (in particular, the vm-PFC) is classically thought to inhibit amygdala activity and reduce subjective distress, while the hippocampus plays a role both in the coding of fear memories and also in the regulation of the amygdala. Both the amygdala and hippocampus are identified as regions of interest in PTSD due to their role in processing and coordination of traumatic memories and perception.^47,48^ They play important roles in emotionally neutral neurocognitive performance.^49^

Our studies showed impaired neurocognition and functioning of PTSD subjects compatible with the possible effects of neuroprogression. While one study showed that impairment in overall intelligence scores and visual memory was negatively associated with illness duration,^40^ two other studies showed that for those individuals who maintain high intensity of symptoms or worsening symptoms, there was greater impairment of verbal memory,^34^ facial recognition^34^ and functioning (physical, psychological, social, and environmental).^12^

Our second conclusion is that, given the high variability of findings within these studies of limited sample size, it seems that having a diagnosis of PTSD at one timepoint does not necessarily predict brain changes at a later timepoint. However, symptom severity and improvement predicted brain changes.^30,34^ While it is difficult to draw conclusions about recovery from PTSD in this kind of research framework, we believe that some of the variability in findings may stem from patients who recovered from PTSD in the interim. The variability of the findings may also be a reflection of the different phenotypic presentations that are grouped within the same diagnosis of PTSD.^50^ Due to this heterogeneity, research on PTSD may reflect specific groups of patients.

McFarlane et al.^51^ developed a model of clinical stages of PTSD, relating different phenotypic presentations to neurobiological changes. According to these authors, time is a critical dimension in dissecting the interplay of the matrix of biological phenomena that have been examined in PTSD. Patients who are at more advanced clinical stages may have: decreased anterior cingulate and hippocampal volume, high allostatic load, high levels of inflammation, medical comorbidities, and entrenched sensitization of a range of neurobiological systems.^51^ Future studies with participants at advanced stages may provide a better understanding of the neuroprogression model.

Another point to consider was the high comorbidity of PTSD with MDD in some studies that showed progressive changes in brain structures.^30,32,35,39^ Among the psychiatric disorders, major depressive disorder (MDD) is highly comorbid with PTSD.^52^ PTSD and MDD comorbidity is associated with greater negative sequelae for individuals with both disorders in comparison to those with either disorder alone, including greater psychological distress,^53^ more severe PTSD,^53^ and a significantly elevated risk of persistent PTSD symptoms.^54^

Our third conclusion is that there has been insufficient study of inflammation in PTSD to correlate this specific marker of neuroprogression with brain changes. A total of 4 studies assessed inflammatory markers: interleukin-1β (IL-1β), interleukin-2 receptor (IL-2R), interleukin-6 (IL-6), interferon-γ (INF-γ), and tumor necrosis factor (TNF-α). Only interleukin IL-1β had an inverse correlation with illness duration,^41,43^ but more studies are needed to correlate it as a specific marker of neuroprogression. Our prediction is that, within the framework of symptom decrement predicting brain changes, identification of ongoing inflammation would further separate out those who will show brain changes at the second timepoint versus those who will not.

A recent review showed that one possible mechanism for the associations between PTSD, inflammation, and oxidative stress is via chronic and repeated activation of the hypothalamic-pituitary-adrenal (HPA) axis, which occurs during re-experiencing of the trauma. Such activation has been identified as a mechanism of stress-related damage to the brain.^27^ Damage to brain regions associated with stress reduction such as the PFC and hippocampus, after this insult, reduces the individual’s adaptive ability to cope with these stresses successfully, constituting the allostatic load. This increases the likelihood that encountering another stressor will produce further excessive stress and immunological response – and the cycle continues.^24^ This closely resembles the theory of neuroprogression in bipolar disorder; bipolar disorder and PTSD have significant comorbidity as well as similarities in their underlying biological phenomena.^55^

An important strength of our systematic review was the search strategy. We used a range of databases. Our study also has some limitations. We were only able to identify a small number of studies that met our inclusion criteria, limiting the extent of our conclusions and indicating the need for further research in this field. Additionally, some of these studies had cross-sectional designs and small sample sizes. In summary, it seems that PTSD is associated with neuroprogression in at least some cases. However, more studies are needed in order to clarify the role of clinical progression in PTSD. Future longitudinal studies examining the mechanism of how traumatic events are linked to cognitive decline and brain changes might offer new insight into how to treat PTSD and prevent pernicious outcomes. Interventions that address neurocognitive decline could potentially be useful in patients with PTSD.
