# Supplementary material for: Neuroprogression in post-traumatic stress disorder: a systematic review
Source: Trends Psychiatry Psychother. 2021 Oct 22;43(3):167–76. doi: 10.47626/2237-6089-2020-0099 (PMC8638708; doi:10.47626/2237-6089-2020-0099)
Supplement: Supplementary file 1 [file 2238-0019-trends-43-03-0167-suppl01.pdf]

http://dx.doi.org/10.47626/2237-6089-2020-0099

Table S1 - Study quality assessment

**Author**	**Selection**	**Comparability**	**Outcome**
Cohort studies			
Heyn et al.[30] (2019)	***	**	**
Yoon et al.[31] (2016)	***	*	**
Bryant et al.[12] (2016)	****		***
Cardenas et al.[34] (2011)	**		**
Vidovic et al.[42] (2011)	**		**
Hakamata et al.[36] (2007)	****	**	***
Bonne et al.[38] (2001)	****	*	**
Cross-sectional studies			
Keding & Herringa[32] (2015)	****	**	***
Chao et al.[33] (2014)	**	**	***
Felmingham et al.[35] (2009)	***	**	***
Emdad et al.[40] (2005)	****	**	***
Villarreal et al.[37] (2002)	***	*	***
De Bellis et al.[39] (1999)	****	**	***
Spivak et al.[43] (1997)	***	*	***
